# Prediction of mortality in cancer patients with COVID-19 using machine learning methods

**DOI:** 10.1097/MD.0000000000045439

**Published:** 2025-10-24

**Authors:** Arzu Babacan

**Affiliations:** a Ankara Dr. Abdurrahman Yurtaslan Oncology Training and Research Hospital, Emergency Service, Ankara, Turkey.

**Keywords:** artificial intelligence, cancer, COVID-19, mortality

## Abstract

This study aimed to predict mortality in cancer patients diagnosed with COVID-19 using machine learning (ML) algorithms and identify the clinical and laboratory parameters associated with mortality. Demographic, clinical, and laboratory data of cancer patients diagnosed with COVID-19 in the emergency service of Dr Abdurrahman Yurtaslan Ankara Oncology Training and Research Hospital were used. Seven ML algorithms, including decision tree, random forest, k-nearest neighbor, Naïve Bayes, eXtreme Gradient Boosting, Adaptive Boosting (AdaBoost), and support vector machines, were used to calculate the mortality risk of patients. Data balancing was achieved using the synthetic minority oversampling technique. Special libraries in the Python 3.8 programming language (Phyton Sofware Foundation, Fredericksburg) were used to determine descriptive statistics, model creation, and model measurement. Mortality risk was calculated using clinical, demographic, and laboratory data related to COVID-19. Data from 306 patients with cancer and COVID-19 were analyzed. Of these, 246 survived, and 60 died. The average age of the patients was 62.1, and 53.6% were male. A total of 60.1% of patients had comorbid conditions. 81.4% had solid malignancies, and 18.6% had hematological malignancies. The best prediction model, in terms of performance metrics such as accuracy (85.86%), sensitivity (86.37%), specificity (85.92%), and F1-score (85.83%), was the random forest algorithm, which was found to be superior to other algorithms, and feature importance analysis was performed using this algorithm. In this analysis, the most important clinical and laboratory parameters determining mortality were ferritin, D-dimer, lactate dehydrogenase, lymphocyte count, C-reactive protein, neutrophil count, lactate, neutrophil-to-lymphocyte ratio, shortness of breath, fever, and loss of taste and smell, which were shown to contribute significantly to model performance. Based on these findings, reliable classification models can be developed using ML methods for cancer patients with COVID-19, and decision-support modules can be created to guide clinicians and healthcare professionals in prioritizing patients based on their mortality risk.

## Graphical abstract

**Figure:**
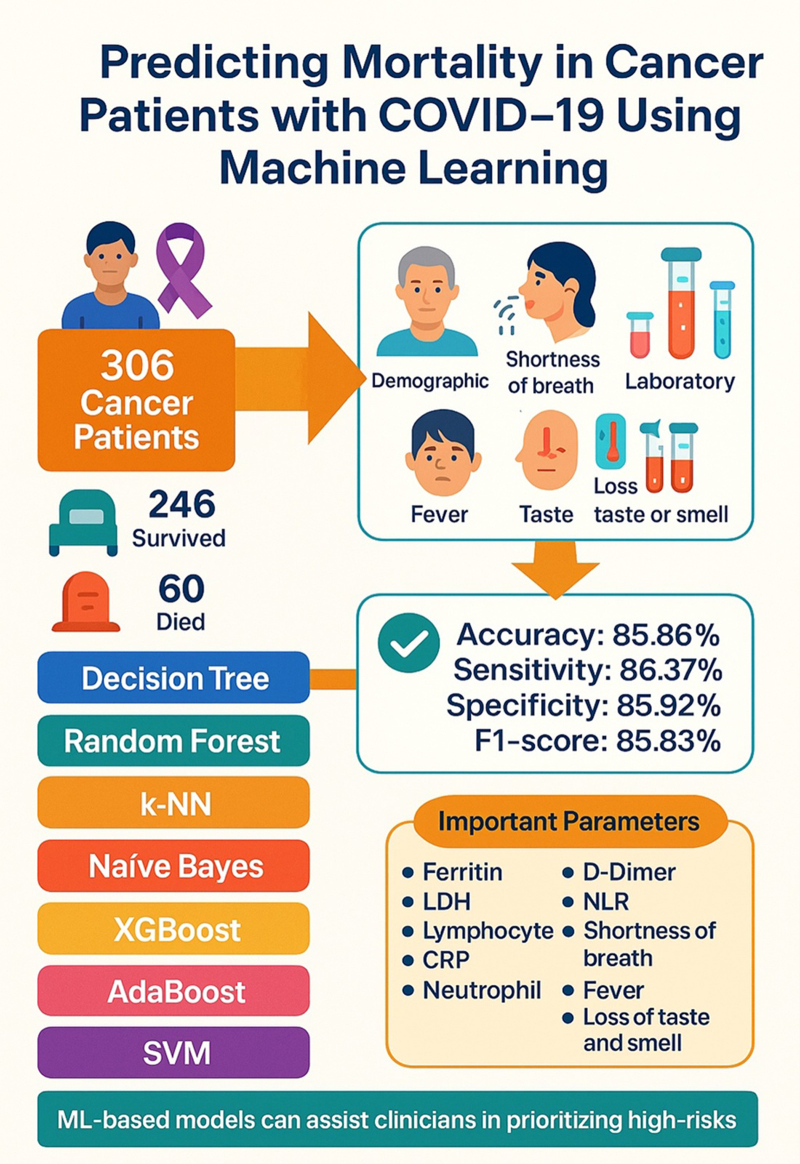

## 1. Introduction

Beginning in December 2019, the world has been affected by the COVID-19 pandemic caused by the SARS-CoV-2 virus. During this period, >656 million confirmed cases and 6.6 million deaths were reported worldwide.^[1]^ COVID-19 infection has a broad clinical spectrum, ranging from asymptomatic cases to severe clinical manifestations such as life-threatening respiratory failure.^[2]^ The most commonly affected organ is the lung, and a severe inflammatory response may progress to multiorgan failure. The most frequently reported symptoms during the pandemic were shortness of breath, fever, fatigue, loss of smell, loss of taste, and muscle pain.^[2]^ Current data have shown that elderly individuals, patients with chronic diseases, and patients receiving cancer treatment are at high risk for COVID-19.^[3]^ Accordingly, research has focused on disease management, identification of risk groups, detection of predictive biomarkers, and the development of effective treatment and prevention strategies. Artificial intelligence (AI) has offered promising solutions in many areas, such as early diagnosis, monitoring of disease spread, contact tracing, identification of risky individuals, and development of drugs and vaccines.^[3]^ China developed AI-based models at the beginning of the outbreak, following individuals’ movements, contacts, and infection risks. Automated diagnostic systems have been created using imaging, blood tests, and clinical data.^[3,4]^ Many studies have been conducted on the successful use of AI for early diagnosis, treatment monitoring, contact tracing, and mortality prediction during the COVID-19 pandemic.^[5]^ Mortality prediction plays a critical role in prioritizing clinical management. In this way, predicting the need for an intensive care unit and managing resources effectively is possible.

Studies have shown that patients with cancer have a higher risk of developing COVID-19 and severe disease than the general population.^[6–8]^ Immunosuppression, frequent hospital admissions, comorbid diseases, and the effect of anticancer therapies complicate the diagnosis and management of COVID-19 in patients with cancer.^[6,7]^ Moreover, the atypical clinical and radiological findings of these patients complicate the diagnostic process. Although there are many studies in the literature on mortality prediction in patients diagnosed with COVID-19, comprehensive ML-based studies on a special immunocompromised, heterogeneous, and high-risk population, such as cancer patients, are limited. Most studies have focused only on specific cancer types and the general population. This study aimed to compare 7 machine learning algorithms (MLAs) to predict mortality in patients with cancer diagnosed with COVID-19. The model with the best performance and the clinical and laboratory parameters that predicted mortality were determined.

## 2. Materials and methods

### 2.1. Study design

This retrospective study was conducted in patients who presented to the emergency service (ES) of Ankara Dr Abdurrahman Yurtaslan Oncology Training and Research Hospital between April 1, 2020, and April 1, 2021. Ethical committee approval was obtained from the University of Health Sciences, Ankara Dr Abdurrahman Yurtaslan Oncology, and Training and Research Hospital. (Date: April 04, 2024, Number: 2024-03/40). The study was conducted in accordance with the principles of the Declaration of Helsinki. A total of 1213 patients over 18 years of age with a diagnosis of solid or hematologic malignancy who presented to the ES with COVID-19 symptoms and underwent reverse transcriptase polymerase chain reaction (RT-PCR) testing of upper or lower respiratory tract specimens for diagnostic evaluation were retrospectively evaluated. Patients with negative RT-PCR results, those admitted to the ES with a positive test result, those with cardiac arrest, and cancer patients under follow-up without treatment were excluded from the study.

Three hundred and six cancer patients receiving active cancer treatment and positive RT-PCR tests were included in the analysis. Demographic information, complaints, malignancy types (solid/hematologic), laboratory parameters (hemogram, biochemistry, neutrophil/lymphocyte ratio [NLR], procalcitonin [PRC], C-reactive protein [CRP], D-dimer, fibrinogen, ferritin, cardiac enzymes, blood gas, lactate dehydrogenase [LDH], and patient outcomes [discharge, hospitalization, referral to another hospital, and death]) were obtained from hospital information management system records and ES patient files. Patients diagnosed with COVID-19 were divided into 2 groups, survivors and non-survivors, and a comparative analysis was performed.

### 2.2. Statistical analysis

Statistical analyses were performed using SPSS version 26 (IBM Co., Armonk). Numerical variables were created using the median with minimum and maximum values. Mann–Whitney *U* and Wilcoxon rank sum tests were used for the analysis of repeated variables and comparisons between the groups, respectively. Chi-square or Fisher’s exact tests were used to compare categorical data. Statistical significance was set at *P* < .05.

### 2.3. Modeling

MLAs are increasingly used in healthcare and are considered promising tools for predicting the course of diseases and performing risk analysis by processing multidimensional datasets. However, problems such as class imbalance and missing data, which are common in medical data, can negatively affect the accuracy and reliability of these algorithms.^[9]^ In this study, data from 306 patients with COVID-19 were analyzed. Of these patients, 246 survived and 60 died. There was no missing data in this dataset. All demographic, clinical, and laboratory parameters were obtained and used in analyses. In our dataset, we observed a significant imbalance between the “survivor” and “dead” groups. Because the significant imbalance between the groups may cause MLAs to be biased toward the dominant class (survivors) and misclassify the minority class (deads), the synthetic minority oversampling technique (SMOTE) was applied to overcome this issue.^[9]^ SMOTE generates synthetic samples by interpolating between observations belonging to the minority class and their nearest neighbors, thereby reducing the imbalance between classes. This technique balances the dataset with 246 samples for both classes. Therefore, the models were trained using an unbiased and balanced dataset. For mortality prediction, 7 MLAs were applied: decision tree (DT), random forest (RF), k-nearest neighbors, Naive Bayes, eXtreme Gradient Boosting (XGBoost), Adaptive Boosting (AdaBoost), and support vector machines (SVM). Hyperparameter optimization of the models was performed using the Grid Search method. The best parameter combinations were selected based on cross-validation performance.

The dataset was divided into 2 subsets to train and evaluate the models: 80% for training and 20% for testing. Ten-fold stratified cross-validation was applied to improve the model performance and generalizability. The performance of the models was assessed based on 4 main metrics: accuracy, precision, recall, and F1 score.

## 3. Results

The records of 1.213 cancer patients were assessed, and 306 patients were included in the final analysis. The median age of the COVID-19-positive patients was 62.1 years; 53.6% were male and 47.4% were female. Cardiovascular disease (33.0%) and DM (19.6%) were the most common comorbidities. Among patients with solid malignancies, 28.5% had gastrointestinal, 20.1% had breast, 19.7% had genitourinary, and 16.9% had lung cancer. Among the patients with hematologic malignancies, 38.6% had leukemia and 31.6% had lymphoma. The metastasis rate was 58.5%. The most common organs of metastases were the lungs (40.2%) and liver (33.5%). Of the patients, 50.3% (n = 154) had received anticancer treatment within the previous month. The most common symptoms were fatigue (79.4%), shortness of breath (60.1%), fever (53.4%), and cough (41.5%). Of the 306 patients diagnosed with COVID-19, 80.4% survived and 19.6% died.

The median age of the non-survivor patients was 65.7 years, and the most common comorbidity was cardiovascular disease (51.7%). The most common cancers were lung cancer (20%), hematologic and gastrointestinal cancers (15%), and breast cancer (13.3%). Lung metastases were present in 40% of patients. Fifty-six percent had received anticancer treatment in the previous month.

The COVID-19-related symptoms were similar to those observed in the general population. White blood cell, neutrophil, NLR, blood urinary nitrogen, ferritin, fibrinogen, LDH, CRP, D-dimer, troponin, PCR, and lactate levels were higher, whereas platelet, lymphocyte, total protein, and albumin levels were lower in the nonsurvivor group than in the living group. The demographic and clinical data of the patients are presented in Table 1, and the laboratory parameters are presented in Table 2.

**Table 1:** Demographic and clinical data of the patients.

	Total (n = 306)	Survivors (n = 246)	Non-survivors (n = 60)
	N/% or median (min–max)		
Age	62.1 (20–90)	61.2 (20–90)	65.7 (35–85)
Gender			
Male	164 (53.6)	129 (52.4)	35 (58.3)
Female	142 (46.4)	117 (47.6)	25 (41.7)
Comorbidity	184 (60.1)	140 (56.9)	44 (73.3)
Type of malignancy			
Hematologic	57 (18.6)	48 (19.5)	9 (15.0)
Solid	249 (81.4)	198 (80.5)	51 (85.0)
Symptoms			
Shortness of breath	184 (60.1)	141 (57.3)	43 (71.7)
Cough	127 (41.5)	99 (40.3)	28 (46.7)
Fever	163 (53.3)	135 (54.9)	28 (46.7)
Fatigue	243 (79.4)	193 (78.5)	50 (83.3)
Muscle pain	74 (24.2)	62 (25.2)	12 (20.0)
Abdominal pain	26 (8.5)	20 (8.1)	6 (10.0)
Diarrhea	29 (9.5)	21 (8.5)	8 (13.3)
Loss of taste and/or smell	7 (2.3)	5 (2.0)	2 (3.3)
General condition disorder	6 (2.0)	6 (2.4)	0 (0.0)
Headache	55 (18.0)	45 (18.3)	10 (16.7)
Sore throat	12 (3.9)	2 (0.8)	10 (16.7)

**Table 2:** Laboratory parameters of the patients.

	Total (n = 306)	Survivors (n = 246)	Non-survivors (n = 60)
Laboratory parameter	Median (minimum–maximum)		
WBC (×10^3^/mm^3^)	12,5 (0.1–297.0)	11.1 (0.1–185.7)	18.1 (0.2–297.0)
Hgb (gr/dL)	10.7 (4.6–17.6)	10.7 (4.6–17.6)	10.6 (5.2–17.3)
Platelet (×10^3^/mm^3^)	212.7 (2.0–850.0)	215.9 (7.0–860.0)	199.3 (2.0–594.0,)
Neutrophile (×10^3^/mm^3^)	8.1 (0.01–61.0)	7.8 (0.01–61.0)	9.3 (0.01–58.9)
Lymphocyte (×10^3^/mm^3^)	0.9 (0.01–5.9)	0.9 (0.06–5.9)	0.9 (0.01–3.7)
NLR	12.8 (0.1–112.0)	12.7 (0.1–112.0)	13.4 (0.1–80.0)”
BUN (mg/dL)	26.8 (3.9–184.4)	24.7 (3.9–184.4)	35.6 (4.4–151.0)
D-dimer (ng/mL)	3014.7 (190.0–4973.0)	2876.8 (190.0–35,200.0)	3580.3 (300.0–4973.0)
Total protein (g/dL)	55.6 (30.4–104.0)	56.1 (30.4–104.0)	53.8 (31.4–76.0)
Albumin (g/L)	29.7 (7.1–72.0)	30.2 (7.1–72.0)	27.4 (17.7–43.0)
Ferritin (ng/mL)	870.3 (14.0–16,687.0)	797.9 (14.0–16,687.0)	1166.9 (96.0–6352.0)
Fibrinjen (mg/dL)	358.2 (0.0–900.0)	357.1 (0.0–786.0)	362.6 (0.0–900.0)
LDH (IU/L)	409.7 (42.0–9624.0)	386.7 (42.0–3159.0)	504.0 (107.0–9624.0)
CRP (mg/dL)	131.8 (0.5–571.0)	129.7 (0.5–571.0)	140.5 (4.0–339.0)
Procalcitonin (ng/mL)	5.4 (0.0–237.0)	5.0 (0.0–75.0)	5.4 (0.0–237.0)
Troponin (ng/mL)	89.4 (0.0–5454.0)	84.9 (0.0–2853.0)	107.7 (0.0–5454.0)
Lactate (mmol/L)	2.4 (0.18–17.7)	2.4 (0.2–17.7)	2.5 (0.7–7.9)

BUN = blood urinary nitrogen, CRP = C-reactive protein, LDH = lactate dehydrogenase, NLR = Neutrophile-lymphocyte ratio, WBC = white blood cell.

In the non-survivor group, symptoms such as shortness of breath, fever, and loss of taste and/or smell on admission were significantly associated with mortality (*P* < .005). Among the laboratory parameters, elevated neutrophil, NLR, D-dimer, ferritin, LDH, CRP, and lactate levels, and low lymphocyte counts were associated with mortality (*P* < .05). Clinical and laboratory parameters associated with mortality are shown in Table 3.

**Table 3:** Clinical and laboratory parameters associated with mortality.

Variables	*P*-value
Age	.088
Gender	.126
Comorbid disease	.213
Malignity	.072
Shortness of breath	**<.01**
Cough	.062
Fever	**<.01**
Fatigue	.095
Muscle pain	.058
Abdominal pain	.069
Diarrhea	.111
Loss of taste and/or smell	**<.01**
General condition disorder	.076
Headache	.162
Throat Pain	.105
WBC	.082
Haemoglobin	.093
Platelet	.145
Neutrophile	**<.01**
Lymphocyte	**<.01**
NLR	**<.01**
BUN	.129
D-dimer	**<.01**
Total protein	.261
Albumin	.066
Ferritin	**<.01**
Fibrinogen	.089
LDH	**<.01**
CRP	**<.01**
Procalcitonin	.102
Troponin	.054
Laktat	**<.01**

Significant *P*-values are shown in bold.

BUN = blood urinary nitrogen, CRP = C-reactive protein, LDH = lactate dehydrogenase, NLR = neutrophile-lymphocyte ratio, WBC = white blood cell.

Table 4 presents the hyperparameter ranges of machine learning models and the best parameter values found using the Grid Search method. This optimization model was implemented to achieve the best performance.

**Table 4:** Hyperparameter ranges of machine learning models.

Model	Hyperparameters	Range	Best parameters
k-nearest neighbors (k-NN)	n_neighbors	[3, 5, 7, 9]	5
	weights	[“uniform,” “distance”]	“distance”
	metric	[“euclidean,” “manhattan”]	“manhattan”
Naive Bayes	var_smoothing	[1e−9, 1e−8, 1e−7, 1e−6]	1.00E−08
Decision tree	max_depth	[3, 5, 10, None]	10
	min_samples_split	[2, 5, 10]	5
	criterion	[“gini,” “entropy”]	“entropy”
SVM	C	[0.1, 1, 10]	10
	kernel	[“linear,” “rbf”]	“rbf”
	gamma	[“scale,” “auto”]	“scale”
AdaBoost	n_estimators	[50, 100, 200]	100
	learning_rate	[0.01, 0.1, 1.0]	0.1
XGBoost	n_estimators	[100, 200]	200
	learning_rate	[0.01, 0.1]	0.1
	max_depth	[3, 5]	5
	subsample	[0.8, 1.0]	1.0
Random forest	n_estimators	[100, 200]	200
	max_depth	[None, 10, 20]	20
	min_samples_split	[2, 5]	2
	criterion	[“gini,” “entropy”]	“gini”

AdaBoost = Adaptive Boosting, SVM = support vector machines, XGBoost = eXtreme Gradient Boosting.

The SMOTE technique was applied because of class imbalance in the dataset, and the non-survivor and survivor groups were equalized. The data distributions before and after SMOTE are shown in Table 5. After data equalization, the performances of 7 different MLAs were evaluated using accuracy, precision, recall, and F1-score metrics. Table 6 shows a comparison of the performance of the algorithms before and after SMOTE. Before SMOTE, the performance of all algorithms in classifying the non-survivor group was found to be low, whereas significant performance improvement was observed in all metrics after SMOTE application. The RF algorithm achieved the highest performance, with an accuracy of 85.86%, a precision of 86.37%, a sensitivity of 85.92%, and an F1-score of 85.83%. The XGBoost and AdaBoost algorithms also exhibited high performance.

**Table 5:** The results of the synthetic minority over-sampling technique.

	Before (data imbalance)	After (balancing with SMOTE)
Total patients	306	492
Survivors	246	246
Non-survivors	60	246

SMOTE = synthetic minority oversampling technique.

**Table 6:** Performance of machine learning algorithms before and after performing the synthetic minority over-sampling technique.

Algorithm		Accuracy	Precision	Recall	F1-score
K-NN	Without SMOTE	74.19 ± 1.8	55.95 ± 2.2	52.98 ± 1.65	52.31 ± 1.45
	With SMOTE	76.82 ± 1.6	74.65 ± 2.0	73.78 ± 1.55	73.44 ± 1.72
Naive Bayes	Without SMOTE	61.29 ± 1.7	49.75 ± 1.2	49.7 ± 1.72	49.46 ± 2.2
	With SMOTE	70.71 ± 1.9	71.79 ± 1.4	70.82 ± 1.92	70.41 ± 2.4
Decision tree	Without SMOTE	69.3 ± 2.6	57.09 ± 2.12	57.44 ± 2.36	57.24 ± 1.8
	With SMOTE	71.72 ± 2.8	72.04 ± 2.45	71.78 ± 1.15	71.65 ± 1.62
Support vector machine	Without SMOTE	66.66 ± 1.41	66.73 ± 1.53	66.93 ± 1.58	66.65 ± 2.64
	With SMOTE	75.76 ± 2.35	75.92 ± 2.42	75.79 ± 2.63	75.74 ± 2.71
AdaBoost	Without SMOTE	70.97 ± 2.05	56.66 ± 2.02	55.95 ± 2.01	56.2 ± 2.26
	With SMOTE	79.79 ± 2.42	79.98 ± 2.54	79.84 ± 2.18	79.78 ± 2.14
XGBoost	Without SMOTE	79.03 ± 1.86	69.42 ± 1.83	66.22 ± 1.96	67.43 ± 1.71
	With SMOTE	83.84 ± 2.22	84.71 ± 2.38	83.92 ± 2.25	83.76 ± 2.28
Random forest	Without SMOTE	77.42 ± 2.63	65.18 ± 2.55	57.59 ± 2.4	58.27 ± 2.8
	With SMOTE	85.86 ± 2.48	86.37 ± 2.29	85.92 ± 2.12	85.83 ± 2.63

AdaBoost = Adaptive Boosting, k-NN = k-nearest neighbors, SMOTE = synthetic minority oversampling technique, XGBoost = eXtreme Gradient Boosting.

**Figure 1.**
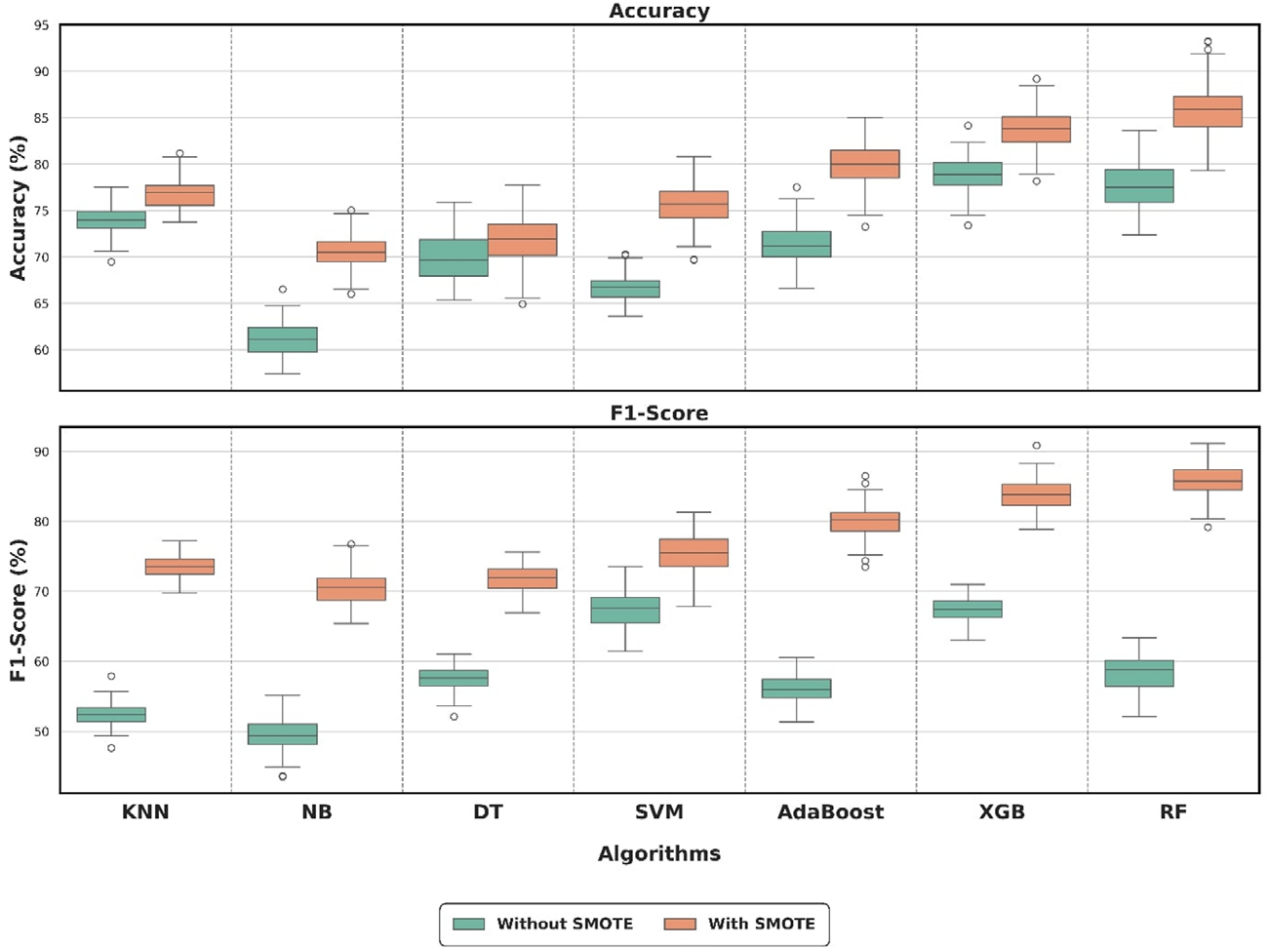
Box plots of ML algorithms before and after SMOTE. The line within the box represents the median, the box shows the interquartile range (25th–75th percentile), and the whiskers indicate minimum and maximum values. Error bars represent ± SD. ML = machine learning, SMOTE = synthetic minority oversampling technique.

The box-plot graphs in Figure 1 illustrate the changes in MLA performance. Although the performance values of the algorithms before SMOTE were low and their distributions were broad, a significant increase in metrics and narrowing of distributions was observed after SMOTE. In particular, the RF and XGBoost algorithms were found to have stable and high success rates in terms of the metric values. To examine the effect of class imbalance in the dataset on modeling performance, the performances of 7 different classification algorithms based on accuracy and F1 score before and after the SMOTE method were compared using 10-fold stratified cross-validation. Stratified cross-validation improves consistency in model evaluation by maintaining the class proportions at each layer. The differences in performance before and after SMOTE were not normally distributed; therefore, the nonparametric Wilcoxon signed-rank test was used. The results of the tests are summarized in Table 7.

**Table 7:** The results of the Wilcoxon signed-rank tests.

Model	Average accuracy difference (%)	*P*-value	Average F1 score difference (%)	*P*-value
k-NN	+2.63	**.004**	+21.13	**.001**
Naive Bayes	+9.42	**<.001**	+20.95	**<.001**
Decision tree	+2.42	**.01**	+14.41	**.002**
SVM	+9.10	**<.001**	+9.09	**.001**
AdaBoost	+8.82	**<.001**	+23.58	**<.001**
XGBoost	+4.81	**.002**	+16.33	**.001**
RF	+8.44	**<.001**	+27.56	**<.001**

Significant *P*-values are shown in bold.

AdaBoost = Adaptive Boosting, k-NN = k-nearest neighbors, RF = random forest, SVM = support vector machines, XGBoost = eXtreme Gradient Boosting.

SMOTE significantly increased the accuracy and F1 scores of all models. As shown in Table 5, significant increases in accuracy and F1 scores were observed in all models after SMOTE application. Although the gains in accuracy were modest, the differences in F1 scores were particularly noticeable. This suggests that SMOTE enables the model to learn the minority class better by replicating minority class instances, thus improving precision and accuracy. For example, the RF model improved its average F1 score by approximately 27.56% after SMOTE, whereas AdaBoost showed a significant improvement of 23.58%. Algorithms such as k-nearest neighbors and DT also exhibited significant but lower performance gains. These results indicate that SMOTE is an effective method for class imbalance problems and improves model performance when used with different algorithms.

**Figure 2.**
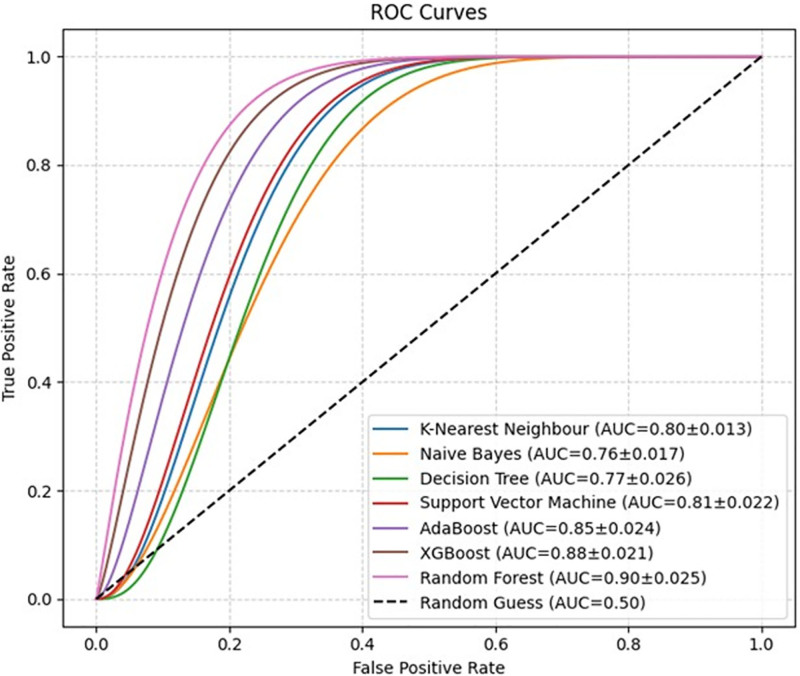
ROC curves with corresponding AUC values are presented. Both the mean AUC scores and their standard deviations are indicated, along with 95% confidence intervals. AUC = area under the curve, ROC = receiver operating characteristic.

After applying the SMOTE technique, the classification success of the MLAs was compared with that of the receiver operating characteristic graph. According to the results shown in Figure 2, the model with the highest area under the curve (AUC) value was the RF (AUC: 0.90 ± 0.025). The XGBoost model showed similarly high performance (AUC: 0.88 ± 0.021). The AdaBoost model achieved the third-best result (AUC = 0.85 ± 0.024). Among the simpler models, KNN (AUC 0.80 ± 0.013) and SVM (AUC 0.81 ± 0.022) also achieved satisfactory results, but Naïve Bayes (AUC 0.76 ± 0.017) and DT (AUC 0.77 ± 0.026) showed lower performances than the others. These results suggest that ensemble-based algorithms (RF, XGBoost, and AdaBoost) provide superior overall performance for the dataset. Furthermore, the AUC values revealed that the models were successful in terms of overall discriminative power.

**Figure 3.**
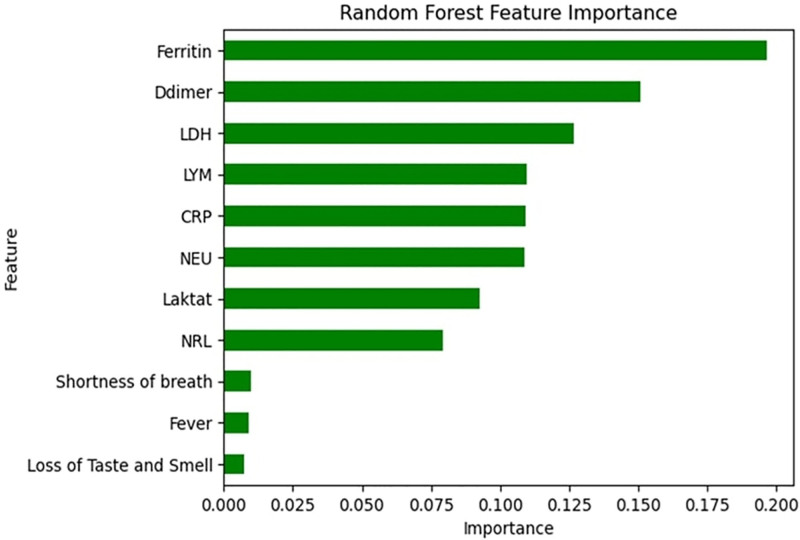
Feature importance analysis of the RF algorithm. The *Y*-axis lists clinical and laboratory parameters, and the *X*-axis shows their relative importance scores. RF = random forest.

**Figure 4.**
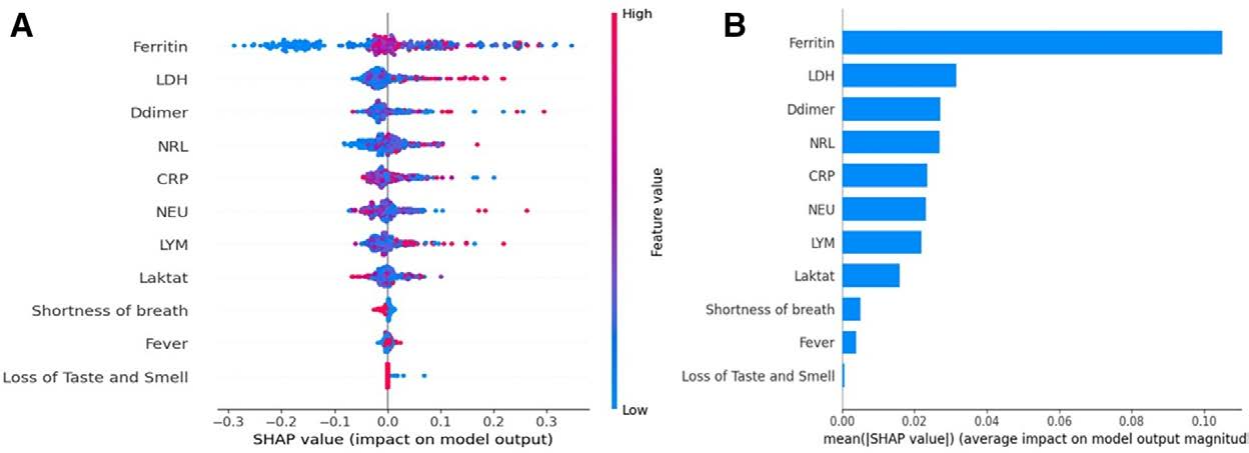
SHAP analysis results. (A) SHAP Bee-swarm plot. Each dot represents an instance, with the *Y*-axis listing features and the *X*-axis showing SHAP values. Red dots indicate high feature values, which increase mortality risk, while blue dots represent low values, reducing the risk. The spread of dots shows feature variability. (B) SHAP global feature importance plot. Features are ranked by their overall contribution to mortality risk prediction, with the most important features at the top. SHAP = SHapley Additive exPlanations.

The RF classification method developed by Breiman et al^[10]^ provides differentiation by extracting small amounts of information from the dataset, requiring minimal preprocessing. It has been reported to be successful in studies on unstable datasets. Furthermore, the algorithm’s ability to generate feature-based importance rankings contributed to the identification of clinically relevant biomarkers. In our research, a feature importance analysis was also performed using the RF algorithm, which achieved the highest prediction rate. In this analysis, laboratory parameters that were determinants of mortality included ferritin, D-dimer, LDH, lymphocyte count, CRP, neutrophil count, lactate, and NLR. Clinical parameters, such as shortness of breath, fever, and loss of taste and smell, also contributed significantly to model performance. The identification of such biomarkers not only improves the predictive power of the models but also provides valuable information for clinical decision-making. The feature-importance analysis is shown in Figure 3. To further substantiate the importance of this feature and enhance the transparency of the model’s decision-making process, SHAP (SHapley Additive exPlanations) analysis was employed. SHAP analysis provides a detailed breakdown of how each feature contributes to the model’s prediction of mortality risk. The results of the SHAP analysis are presented in Figure 4. By showing the Bee-swarm plots (Fig. 4A) and overall feature importance (Fig. 4B), the contributions of individual features and their relative importance across the entire dataset are more clearly revealed. According to the results, high levels of ferritin, LDH, D-dimer, NLR, CRP, neutrophils, and lactate were identified as the strongest risk factors significantly increasing mortality, while a low lymphocyte count was associated with a higher mortality risk. Additionally, clinical symptoms such as shortness of breath, fever, and loss of taste and smell were found to be strongly predictive of increased mortality risk.

## 4. Discussion

The rapid spread of COVID-19, the emergence of new variants, and changing clinical findings over time have led to the disease being recognized as one of the most devastating global health crises of the 21st century. Indeed, the World Health Organization has emphasized that the number of active COVID-19 cases and mortality rates are higher than reported rates, according to 2024 data.^[11]^ Studies conducted on cancer patients in the literature have found different rates of positivity ranging from 7.3% to 64.0% and different mortality rates varying between 18% and 41.3%.^[8,12,13]^ In our study, the COVID-19 positivity rate was 45.3%, and the mortality rate was 19.6%, which is consistent with the literature. Many studies have emphasized that advanced age, male gender, and accompanying cardiovascular and metabolic diseases increase the mortality rate in infected patients with cancer.^[14–18]^ Lee et al^[19]^ reported that mortality associated with COVID-19 varies according to cancer type and that not all cancer patients have the same risk profile. Some studies have found that mortality rates in patients with lung and hematological cancers are significantly higher than in patients with other types of cancer.^[19–22]^ The most common symptoms are fever, cough, shortness of breath, and fatigue, similar to those in the general population.^[17,19,20]^ Dyspnea in COVID-19 disease is accepted as a critical prognostic factor because it predicts serious clinical outcomes such as lung involvement and hypoxia.^[23,24]^ Indeed, Song et al^[20]^ found that dyspnea and fever were prominent in cancer patients who died, and that dyspnea had a significant impact on disease prognosis. It has been found that high levels of D-dimer, CRP, LDH, PRC, lactate, NLR, ferritin, neutrophils, and low levels of lymphocytes, albumin, and platelets are associated with hospitalization and mortality.^[16–18,20,25]^ In this study, most of the patients who died were elderly, male, with cardiovascular disease, lung cancer, shortness of breath, and fever. Neutrophils, NLR, D-dimer, ferritin, LDH, CRP, elevated lactate, and low lymphocyte counts were determined as biomarkers associated with mortality. Our results align with those of previous studies.

Imbalanced datasets can often cause seemingly insurmountable obstacles to model performance in ML. In many standard ML algorithms, classes are expected to be represented similarly in the data distribution. Class imbalance is one of the dataset characteristics that negatively affects the performance of ML algorithms.^[9]^ In imbalanced datasets, there are often datasets where one class is significantly more prominent than the others. When dealing with imbalanced datasets, a decrease in the performance values of classifiers is observed. Imbalance can lead to classifiers being biased toward the majority class, resulting in models that overlook the critical minority cases of interest.^[9,26]^ Research conducted on medical data often encounters imbalanced dataset problems. Therefore, it is important to use data sampling methods (resampling methods, ensemble learning, etc) that balance imbalanced datasets before the classification stage to improve the performance of classifiers. Resampling can add samples from the minority class or remove samples from the majority class to balance the classes. Random oversampling is a simple method that creates new samples for the less common class by multiplying the existing samples. One of the advanced approaches is the SMOTE, which creates new samples by interpolating between existing minority class samples. It adjusts the number of examples in the minority class to be closer to the number of examples in the majority class. The imbalance between the number of examples produced and the number of examples in the majority class is eliminated. The SMOTE algorithm proposed by Chawla et al is among the best algorithms for dealing with imbalanced data sets. The advantage of this method is that there is no data loss.^[9,26]^ However, SMOTE may not fully capture the clinical variability of the original dataset because it generates synthetic samples, which may lead to potential biases. Alternatively, in the ensemble resampling method, small classes are oversampled, and large classes are undersampled.^[27,28]^ The resampling scale is determined according to the ratio of the lowest class count to the highest class count. Multiple ML methods are selected to form the ensemble. This reduces bias and variance, and improves overall prediction performance.^[27,28]^ There are studies in the literature where ensemble learning methods, which combine the predictive capabilities of 2 or more basic learning models, are used in COVID-19 data analysis.^[29,30]^

In the dataset analyzed in our study, the imbalance between the number of survivors and non-survivors was balanced using the SMOTE method, resulting in an equal number of samples for both classes. After applying SMOTE, a significant performance improvement was observed in all algorithms. These results demonstrate that SMOTE effectively resolves the class imbalance issue and significantly improves model accuracy in naturally imbalanced datasets such as medical data. Numerous studies in the literature have utilized the SMOTE method.^[31–34]^

Studies have been conducted to develop models for predicting disease, analyzing risk, predicting deterioration, and predicting mortality using ML algorithms. The number of clinical data sets, the variety of algorithms, and the preferences in selecting appropriate algorithms have resulted in different prediction model studies. In disease prediction, the RF model was used by Assaf et al^[35]^ with an AUC of 0.92, Demiraslan et al^[36]^ used the SMOTE technique in their study and found an AUC of 0.87, while Brinati et al^[37]^ used age, leukocyte, platelet, CRP, AST, ALT, LDH, and neutrophil data in their study and found an AUC of 0.82, emphasizing that it is a successful and reliable model. In the study by Orhanbulucu et al^[31]^ it was observed that the rapidly spreading coronavirus disease could be detected using some samples obtained from the laboratory, thanks to the high success rate (93.3%) of the RF classification algorithm.

In predicting the severity of COVID-19, Kutlu et al^[38]^ used the SMOTE method and the Regression Trees model to obtain an AUC (0.83) prediction result; age, cardiovascular disease, hypertension, and cancer were predicted to be the 4 most important factors. Gök and Olgun^[33]^ found that the RF algorithm achieved an AUC (0.98) accuracy and that LDH, lymphocytes, and CRP were the most relevant biomarkers for mortality with 90% accuracy. In studies on mortality prediction, Aktar et al^[39]^ investigated comorbidities and symptoms that increase the risk of death in COVID-19 patients. Age, gender, COPD, cardiovascular disease, chronic kidney disease, diabetes, malignancy, hypertension, and asthma have been identified as significant risk factors in ML. Moulaei et al^[32]^ reported that the RF algorithm showed the highest performance with an accuracy of 95% and an AUC of 99.02%. Çakmak et al^[40]^ found similar results with RF and DT algorithms and highlighted parameters such as D-dimer, PRC, and ferritin as biomarkers associated with mortality. Booth et al^[41]^ reported that the SVM algorithm yielded high discrimination with an AUC of 0.93. Kourmpanis et al^[42]^ reported that XGBoost was the most accurate algorithm with an AUC of 0.97. Shanbehzadeh et al^[43]^ highlighted the Bayesian network algorithm, and Jamshidi et al^[44]^ highlighted the RF model. Weiser^[45]^ demonstrated that RF achieved the best performance in predicting mortality, with an AUC of 0.98. Dyspnea, cough, and fever have been reported as the strongest clinical predictors associated with poor prognosis in ML-based models in the literature.^[35,46,47]^

In summary, 7 different ML algorithms were compared for the prediction of mortality in COVID-19-infected patients with cancer in this study. The RF model yielded the most successful results in terms of accuracy (85.86%), precision (86.37%), sensitivity (85.92%), F1 score (85.83%), and AUC (0.90). The RF model was also used for “feature importance analysis,” and the most effective laboratory parameters for mortality prediction were determined to be ferritin, D-dimer, LDH, lymphocytes, CRP, neutrophils, lactate, and NLR. Dyspnea, fever, and loss of taste/smell were the most prominent clinical variables. To further improve the interpretability of the model’s predictions and ensure transparency, a SHAP analysis was performed, detailing how each feature contributes to mortality risk prediction. According to the SHAP analysis results, high levels of ferritin, LDH, D-dimer, NLR, CRP, neutrophils, and lactate were identified as the strongest contributors to increased mortality risk, while low lymphocyte counts were associated with increased mortality risk. Clinical symptoms such as dyspnea, fever, and loss of taste and smell also strongly predicted high mortality. This model can provide a valuable guide to clinicians to identify high-risk patients earlier by further improving its clinical interpretability.

RF has become the leading model in this study because of its high accuracy and generalizability. ML-based models developed for mortality prediction in cancer patients infected with COVID-19 are pretty limited. Studies in the literature are generally limited to the general population or specific cancer types. Therefore, this study fills a critical gap by being one of the first to compare ML algorithms for predicting mortality in cancer patients with different types of malignancies.

In this study, the high accuracy rate of the RF algorithm and the identification of predictive parameters can be used in the development of clinical decision support systems. It can make an important contribution to the early diagnosis of high-risk patients and the determination of follow-up and treatment priorities. Such models can contribute to the efficient use of limited resources, thereby improving the quality of care and survival rates.

This study has some limitations. First, this study is a single-center, retrospective analysis. The limited number of cases in the non-survivor group may pose a risk of overfitting in some models. Therefore, model performance should be validated with larger and multicenter datasets. The data only include clinical and laboratory findings at the time of ES admission. Additionally, the SMOTE technique enables the algorithm to learn the minority class more effectively. It should be noted that synthetic data may not fully reflect clinical reality. Finally, the classification models obtained have not yet been prospectively tested in clinical settings. Furthermore, the model’s external validity should be evaluated by testing it on independent and multicenter datasets. Such an external validity assessment will reveal whether the model maintains reliable performance across different patient populations. In future studies, more detailed prognostic information could be obtained by using time-dependent models or survival analysis techniques, rather than relying solely on binary outcome variables.

## 5. Conclusion

Our findings demonstrate that the RF algorithm is an effective predictive model in this patient group, particularly due to its high accuracy and discrimination capacity. The SMOTE technique is an effective method to increase the performance of machine learning techniques in unbalanced distributions frequently encountered in medical data. This model could assist in the early identification of high-risk cancer patients at ES admission and help prioritize those requiring intensive care. Consequently, clinical decision-making could be accelerated, and resource utilization optimized. Further studies are needed to test the usability of these models for real-time patient management decisions.

## Author contributions

**Conceptualization:** Arzu Babacan.

**Data curation:** Arzu Babacan.

**Formal analysis:** Arzu Babacan.

**Funding acquisition:** Arzu Babacan.

**Investigation:** Arzu Babacan.

**Methodology:** Arzu Babacan.

**Project administration:** Arzu Babacan.

**Resources:** Arzu Babacan.

**Software:** Arzu Babacan.

**Validation:** Arzu Babacan.

**Visualization:** Arzu Babacan.

**Writing – original draft:** Arzu Babacan.

**Writing – review & editing:** Arzu Babacan.
